# Future Climate Data from RCP 4.5 and Occurrence of Malaria in Korea

**DOI:** 10.3390/ijerph111010587

**Published:** 2014-10-15

**Authors:** Jaewon Kwak, Huiseong Noh, Soojun Kim, Vijay P. Singh, Seung Jin Hong, Duckgil Kim, Keonhaeng Lee, Narae Kang, Hung Soo Kim

**Affiliations:** 1 Hydroclimatic Statistical Research Group, Centre Eau Terre Environnement, INRS, Québec, QC G1K 9A9, Canada; E-Mail: Jaewon.Kwak@ete.inrs.ca; 2 Department of Civil Engineering, Inha University, Incheon 402-751, Korea; E-Mails: heesung80@hanmail.net (H.N.); hongsst81@gmail.com (S.J.H.); naraeme@naver.com (N.K.); 3 Columbia Water Center, Earth Institute, Columbia University, New York, NY 10027, USA; E-Mail: soojun78@gmail.com; 4 Department of Biological and Agricultural Engineering, Zachry Department of Civil Engineering, Texas A & M University, College Station, TX 77843, USA; E-Mail: vsingh@tamu.edu; 5 Water Environment Research Department, Water Quality Assessment Research Division, National Institute of Environmental Research, Incheon 404-708, Korea; E-Mail: gilkim@korea.kr; 6 Water Resources Research Division, Water Resources and Environment Research Department, Korea Institute of Civil Engineering and Building Technology, Goyang-si, Gyeonggi-do 411-712, Korea; E-Mail: leeggun@kict.re.kr

**Keywords:** malaria, climate change, PCA-regression analysis, climate variable

## Abstract

Since its reappearance at the Military Demarcation Line in 1993, malaria has been occurring annually in Korea. Malaria is regarded as a third grade nationally notifiable disease susceptible to climate change. The objective of this study is to quantify the effect of climatic factors on the occurrence of malaria in Korea and construct a malaria occurrence model for predicting the future trend of malaria under the influence of climate change. Using data from 2001–2011, the effect of time lag between malaria occurrence and mean temperature, relative humidity and total precipitation was investigated using spectral analysis. Also, a principal component regression model was constructed, considering multicollinearity. Future climate data, generated from RCP 4.5 climate change scenario and CNCM3 climate model, was applied to the constructed regression model to simulate future malaria occurrence and analyze the trend of occurrence. Results show an increase in the occurrence of malaria and the shortening of annual time of occurrence in the future.

## 1. Introduction

Malaria is an acute infectious disease caused by plasmodium parasite infection in red blood cells and liver cells. Approximately 300–500 million cases of malaria patients are reported annually, and over a million patients die from this parasitic infection. The World Health Organization (WHO) has designated malaria as one of the six major tropical diseases. Infection through plasmodium vivax and plasmodium falciparum is a cause of more than 95% of reported cases, and in Korea, plasmodium vivax infection is found in the majority of the reported cases. Generally, infectious diseases that use host as a means of infection are highly susceptible to climate which influences interactions within the ecosystem [1]. Mosquito-borne diseases are especially susceptible and it is known that they are greatly influenced by temperature, precipitation, humidity and other factors. Amongst these diseases, malaria is the most well addressed infectious disease and it is also most susceptible to climate change [2]. 

Many studies have been conducted to investigate how malaria is related to climate change and climate factors. Poveda *et al*. [3] conducted a study of malaria and time lag effect of El Nino climate in Columbia, U.S. and Craig *et al*. [4] investigated the relationship between malaria and the probability distribution of climate factors in the Sahara region. Paaijmans *et al*. [5] correlated malaria to mean temperature and analyzed the change in the influence of malaria with daily temperature change [6]. Olson *et al*. [7] analyzed the relationship between malaria and wetland and climate factors. Prediction of malaria using an impulse function computed from the climate factors has been made by Kuhn *et al*. [8], whereas Thomson *et al*. [9] predicted malaria infection through seasonal climate factors. Jhajharia *et al*. [10] investigated the influence of climate on incidences of malaria in the Thar desert of Northwest India and found that climatic variability and rise in temperature were key determinants to the transmission of malaria. 

Current malaria and climate-related investigations have focused on determining the correlation between malaria and climate factors for predicting the occurrence of malaria [11,12,13,14,15]. Since climate change is now accepted as real, changes in the characteristics of malaria in relation to climate change have been investigated [16,17]. For example, malaria’s vulnerability to trends of the past century have been analyzed [2,18,19]. Likewise, changes in the future characteristics of malaria in Africa, the most malaria-vulnerable continent, have been analyzed [20,21,22]. In order to prepare adequately for ongoing global warming, van Lieshout *et al*. [23] predicted fluctuations in malaria incidences according to the SRES climate change scenario. These studies show future trends of malaria and climate change and how the occurrence of malaria infection will be affected by future climate change [16,24,25,26,27,28,29,30,31]. 

The objective of this study is therefore to investigate the occurrence of malaria and its correlation with climatic factors and construct a regression model for predicting malaria occurrences in Korea from climate factors. By simulating future malaria occurrences in Korea, the model can provide basic data for public health agencies. For this study, data on monthly malaria occurrences and climatic factors from 2001–2011 was collected. By applying the climate data generated from a climate change scenario and a climate model to the regression model, future malaria occurrences and their trends were analyzed. 

## 2. Malaria Occurrence in Korea

### 2.1. Trend of Malaria Occurrence

Although it is commonly believed that malaria seems to be decreasing in Korea since 2000 (active duty military service men included), facts point otherwise. For example, 1325 malaria patients were reported in 2005, 2021 cases in 2006, and 2192 cases in 2007, showing an 8.5% increase (Centers for Disease Control & Prevention, [32]). The communication of malaria is caused by female Anopehline [33]. Most mosquitoes that carry malaria show a much more vibrant activity as weather gets warmer [34]. Therefore, in Korea, an increase of malaria infection is strongly related to the rise in temperature caused by climate change [35].

### 2.2. Data Collection

**Figure 1:**
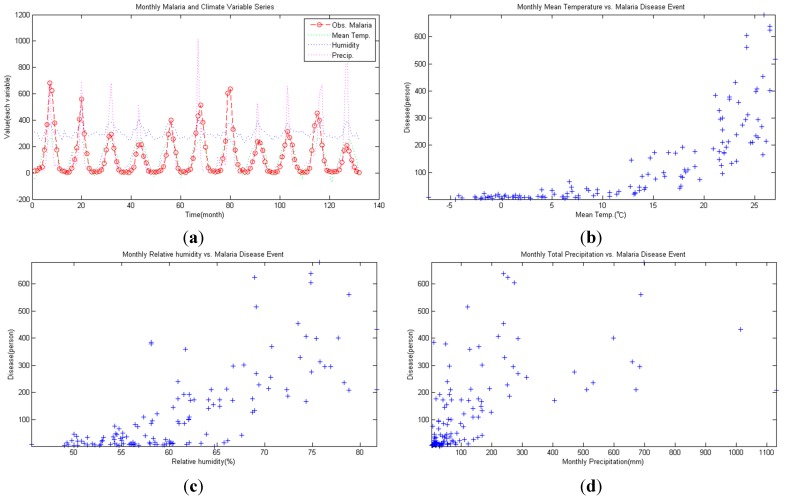
(**a**) Time series of each variable; (**b**) scatter diagram of mean temperature and malaria; (**c**) scatter diagram of relative humidity and malaria; (**d**) scatter diagram of monthly precipitation and malaria.

Jang *et al.* [1] has reported that malaria has the highest infected domestic patients among the diseases showing relevance to climate factors and climate change. Since national infectious disease database has been established, quality data can now be acquired. Monthly data of the designated infectious diseases between 2001 and 2011 from the Center for Disease Control & Prevention was utilized for obtaining data on malaria occurrences. Data from 2001–2008 was used for calibration and data from 2009–2011 was used for verification. For the same period, climate data was obtained from the Seoul Regional Meteorological Observatory. Based on the data from Center for Disease Control & Prevention [32], the highest malaria occurrence in 2008 happened in Gyeonggi province (475 reported cases), followed by Seoul (180 reported cases), and Incheon (92 reported cases). The distribution of malaria was highly concentrated in the Seoul and Gyeonggi areas. Thus, climate variables from the Gyeonggi and Seoul Meteorological Observatories were averaged into one for use in this study. Also, data from weather stations at E.L. 200 m above the sea level may disrupt the average values of regional meteorological factors, so these were excluded from analysis. Meteorological factors included in the analysis were average temperature (°C), relative humidity (%), and precipitation (mm). The collected meteorological and malaria data are shown in Figure 1a, and the scatter diagram of malaria is shown in Figure 1a and for average temperature, relative humidity and precipitation, Figure 1d is referred to. 

## 3. Methodology

This study performs a regression analysis for modeling the correlation between malaria which is the dependent variable and climate variables which are independent variables. While doing so, spectral analysis must be done to reflect the time lag between each variable and the BDS test was done to check the randomness of the time series. Also, the principal components regression should be applied and tested for considering multicollinearity between variables [36,37].

### 3.1. Spectral Analysis

Autospectrum or crossspectrum is a method to explain the distribution of variance of frequency drawn from a single or multiple data [38]. Covariance was estimated using autocovariance for data for time interval t and lag k :

(1) $$Cov_{xx}(k)=\frac{1}{N-k-1}\sum_{t=1}^{N-k}\left(x_{t}-\bar{x}\right)\left(x_{t+k}-\bar{x}\right)$$

where N : Number of data series, t , and Δt : time interval and time step.

Autocorrelation function can be obtained by dividing the autocovariance by $\sigma^{2}$ , variance of x(t) . Spectral function $X_{xx}(f)$ can be obtained by applying the Fourier transform to the autocorrelation function. Spectral analysis would give periodic information of the malaria time series. Cross spectral analysis can also be applied to cross covariance of the x(t) , y(t) times series and can be also used to differentiate the relevant covariance of two time series data set to have lag k . Cross covariance and cross spectrum ($X_{xy}$) of two times series with time interval t can be determined using Equations (2) and (3):

(2) $$Cov_{xy}(k)=\frac{1}{N-k-1}\sum_{t=1}^{N-k}\left(x_{t}-\bar{x}\right)\left(y_{t+k}-\bar{y}\right)$$

(3) $$\left|X_{XY}\right|=\sum_{k=0}^{Maxlag}\left(\frac{Cov_{xy}(k)}{\sigma_{x}\sigma_{y}}\right)\left(k\right)e^{\frac{i2\pi fk}{f_{s}}}\text{ , }f_{s}\text{: sampling frequency}$$

Also, using the cross spectrum on two time series, coherency can be calculated and information about the frequency between the two (see Equation (4)) can be obtained. Coherency between two time series is commonly represented in the 1 – $C_{xy}$ form.

(4) $$C_{xy}=\frac{{\left|X_{xx}\right|}^{2}}{X_{xx}X_{yy}}$$

where $C_{xy}$ : coherency of time series, $X_{xx}$ , and $X_{yy}$ : autospectral density of each time series.

### 3.2. Brock-Dechert-Scheinkman(BDS) Statistic

The BDS statistics is a method to verify a hypothesis that times series complies with the random distribution based on the correlation integral. It is an effective tool to discern between random time series and nonlinear chaos or stochastic system [39,40,41]. Grassberger and Procaccia [42] suggested correlation integral as a method to measure the fractal dimension on deterministic data, and for embedding dimension *m*, computed as

(5) $$C(m,N,r)=\frac{2}{M(M-1)}\sum_{1\leq i<j\leq M}\Theta (r-\left\|{\vec{x}}_{i}-{\vec{x}}_{j}\right\|),\;r>0\;$$

where, $\begin{array}{l} \Theta (a)=0,\;if\;a\leq 0\\ \Theta (a)=1,\;if\;a\geq 0\;. \end{array}$

*N* is the size of the data sets, *M = N – (m – 1)t* is the number of embedded points in *m*-dimensional space, and ‖ ⋅  ‖ denotes the sup-norm. On target data ${\vec{x}}_{i}=(x_{i},\;x_{i+t},\;\cdots ,\;x_{i+(m-1)t}),\;{\vec{x}}_{i}\in R^{m}$ , i=[1,2,⋯,N] , if the supremum norm is less than the *r* value, and C(m,N,r) becomes a fractal value for the target data. If *N* expands to infinity for each *r* value, the fractal dimension within the *r* value can be defined as $C(m,r)=\underset{N\to \infty }{\lim}C(m,N,r)$ . In the case where target data is a stochastic time series with stationary character and also possesses frequency and limit, the value of the corresponding limit can be represented as shown in Equation (6):

(6) $$C(m,r)=\int \int_{X}\Theta (r-\left\|\vec{x}-\vec{y}\right\|)dF(\vec{x})dF(\vec{y}),\;r>0\;$$

Here, if data possesses independent identically distributed characteristics, $\Theta (r-\left\|\vec{x}-\vec{y}\right\|)$ can be represented as $\prod_{k=1}^{m}\Theta (r-\left|x_{k}-y_{k}\right|)$ and Eq. (6) become $C(m,r)=C^{m}(1,r)$ . In this case, $C(m,r)=C^{m}(1,r)$ is an asymptotic normal distribution with an average of 0, and variance $\sigma^{2}$ as follows:

(7) $$\begin{array}{l} \sigma^{2}(m,M,r)/4=m(m-1)C^{2(m-1)}(K-C^{2})+K^{m}-C^{2m}\\ \;+2\sum_{i=1}^{m-1}\left[C^{2i}(K^{m-i}-C^{2(m-i)})-mC^{2(m-i)}(K-C^{2})\right]\; \end{array}$$

Thus, using C(1,r) and the K value, the value for coefficient C can be calculated as

(8) $$K(m,M,r)=\frac{6}{M(M-1)(M-2)}\sum_{1\leq i<j\leq M}[\Theta (r-\left\|{\vec{x}}_{i}-{\vec{x}}_{j}\right\|)\Theta (r-\left\|{\vec{x}}_{i}-{\vec{x}}_{j}\right\|)]\;$$

Thus, under the assumption that time series data is independent identically distributed, where m > 1, the BDS statistic can be represented as

(9) $$BDS(m,M,r)=\frac{\sqrt{M}}{\sigma }[C(m,r)-C^{m}(1,r)]\;$$

The BDS statistic can be an effective tool to determine whether the given time series is random or is a nonlinear system (chaos or stochastic). To utilize the BDS statistic, selection of the m and *r* values is critical. In this study, m was defined as 2≤m≤5 [39], and for r , 0.5σ≤r≤1.5σ (σ = standard deviation) [43].

### 3.3. Principal Components Regression

If there is a correlation between selected independent variables that show multicollinearity during regression analysis, then some of the independent variables may be eliminated or new sets of observed values may be introduced. Also, depending on the situation in the field, correlation itself is also subject to elimination [44]. However, elimination of independent variables is not desirable, considering the information loss. As an alternative, principal component regression (PCR) is often utilized [45,46,47,48]. Principal component regression selects principal components (less than original variable in numbers) and combines them with the regression model through principal components analysis (PCA) by compressing the dimension as suggested by Morison [49]. By estimating the principle component score of independent variables and conducting regression analysis, information loss can be minimalized while avoiding multicollinearity [50].

In principal component regression, the eigenvalue ($\lambda_{i}$) matrix Λ of the correlation coefficient matrix and eigenvector V satisfy the following Equation (10):

(10) $$V^{T}\left(Z^{T}Z\right)V=\Lambda \text{, }V^{T}V=VV^{T}=I$$

Eigenvector is an orthogonal matrix, so $VV^{T}=I$ is valid. When Equation (10) is substituted in the general form of regression model in the matrix form, it can be arranged into a regression model as:

(11) $$y=XVV^{T}\beta +\varepsilon$$

where X : independent variables of i ^th^, and β : coefficients matrix of independent variables, ε : error term.

In Equation (11), XV is a linear combination of independent variables $X=\left[x_{1},x_{2},x_{3}.\ldots ,x_{n}\right]$ and eigenvector X . It is a transformation of independent variable by multiplying the eigenvector so variables would be orthogonal and it is called principal components. Principal components are independent, so the multicollinearity issue is resolved and thus regression analysis can be conducted without loss of information. 

## 4. Modeling of Malaria and Climate Variables

### 4.1. Nonlinear Regression Analysis

**Figure 2:**
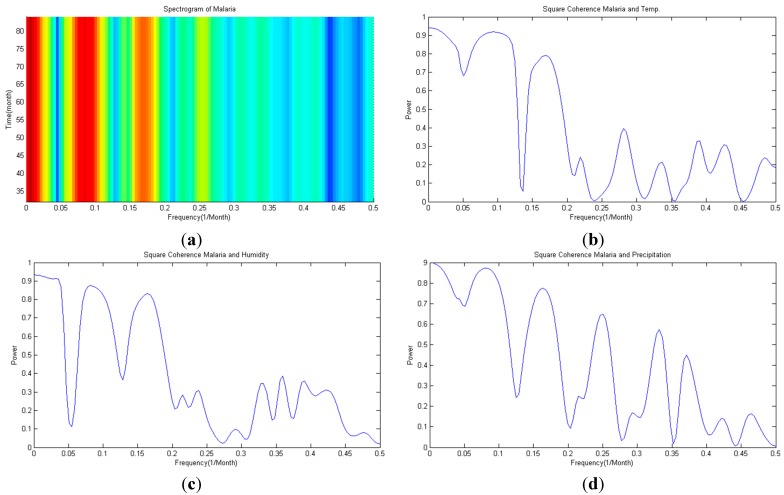
(**a**) Spectrogram of malaria; (**b**) coherency of Temp. and malaria; (**c**) coherency of humidity and malaria; (**d**) coherency of precipitation and malaria.

Regression analysis was conducted on monthly malaria occurrences and climate variables for the period between 2001 and 2008. Nonlinear regression was applied to climate variables and reported malaria occurrence counts to conduct regression analysis. Many recent studies [51,52] that studied malaria in Korea and climate variables considered the time lag effect so it was assumed that there is correlation in accordance with the time lag. So, autospectral analysis is in accordance with Figure 2a, and it also represented coherency analysis between each climate variable and malaria occurrencesin Figure 2b–d. 

As the autospectrum result of malaria shows in Figure 2a, the malaria occurrence time series seems to have a 12 month strong frequency (=0.083) and a 6 month weak frequency (=0.167). Figure 1a confirms that the malaria occurrence series possesses an annual period. Looking at the squared coherency from Figure 2b–d, the frequency of each climate variable and malaria can be defined. Coherency between malaria and average temperature shows a high coherency in 12 and 6 months (see Figure 2b), and the same can be said for humidity (see Figure 2c). Lastly, for malaria and precipitation, high coherency is shown in 12, 6 and 4 month periods. Considering that coherency explains the correlation level exhibited in every time series, each coherency seems to be the time lag effect for respective coherency. Thus, in this study, the time lag effect of respective period was considered when doing regression analysis. 

Also, when data is analyzed, they are assumed random variables and it is assumed that each variable is independent when doing regression analysis. So, tests for randomness and nonlinearity were conducted for the malaria occurrence series. In this research, four commonly used nonparametric tests (Anderson Correlogram, Run Test, Spearman Rank, Correlation Coefficient and Turning point test) that Salas *et al*. [53] suggested and BDS statistics test were used.

**Figure 3:**
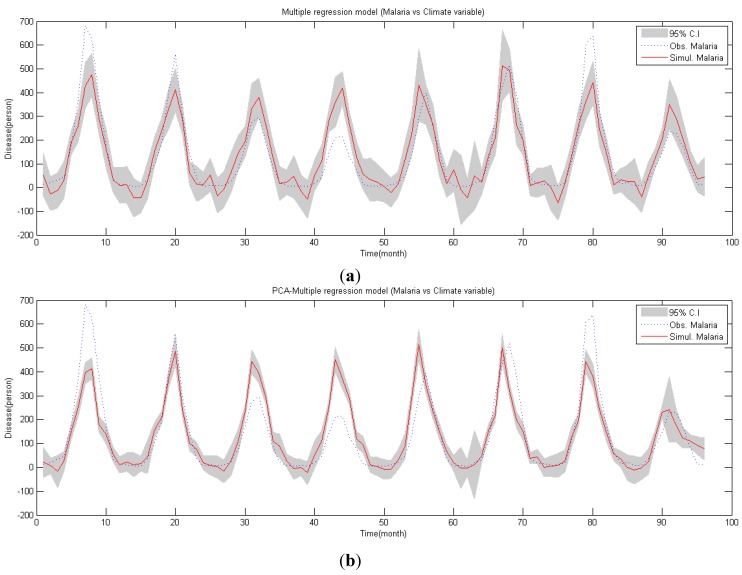
Malaria simulation (2001–2008) (**a**) Malaria simulation by multiple regression; (**b**) malaria simulation by PCA-regression.

The Anderson correlogram and Spearman test judged data as random data as shown in Table 1, but run test and turning point inferred otherwise. Statistics of BDS (m) showed that it exceeded the significance level in every dimension and this shows malaria occurrence series possessed *nonlinear chaotic* or *stochastic property*. Given this, when constructing the regression model for malaria occurrences, regression analysis was conducted with consideration for nonlinearity. Results of multiple regression analysis of malaria occurrences based on nonlinear regression analysis are shown in Figure 3a and Equation (12).

(12) $$\begin{array}{l} y=1164-11.76x_{1}+9.25x_{2}-4.24x_{3}+7.05x_{4}-16.75x_{5}-25.90x_{6}-0.34x_{7}\\ +0.0076x_{8}+0.011x_{9}-0.14x_{10}+1.03x_{1}^{2}-0.39x_{2}^{2}+0.063x_{3}^{2}-0.038x_{4}^{2}\\ +0.11x_{5}^{2}+0.21x_{6}^{2}+0.0005x_{7}^{2}+1.6\times 10^{-5}x_{8}^{2}+1.2\times 10^{-5}x_{9}^{2}+0.00019x_{10}^{2} \end{array}$$

where y : monthly malaria occurrence (person); $x_{1}$ , $x_{2}$ , $x_{3}$ : monthly mean temperature(℃) with 0, 6, 12 month lags; $x_{4}$ , $x_{5}$ , $x_{6}$ : monthly relative humidity(%) with 0, 6, 12 month lags; and $x_{7}$ , $x_{8}$ , $x_{9}$ , $x_{10}$ : monthly total precipitation(mm) with 0, 6, 12, 4 month lags.

**Table 1:** Test statistics of malaria time series (2001–2011).

Test method		Test statistic	95% C. I.	Randomness Check
Run Test		−7.3261	[−1.96, +1.96]	X
Anderson		0.1741	[−1.65. +1.65]	O
Spearman		0.5760	[−1.96, +1.96]	O
Turning Point		−11.9868	[−1.96, +1.96]	X
BDS(2)		10.5150	[−1.96, +1.96]	X
BDS(3)		9.7895	[−1.96, +1.96]	X
BDS(4)		9.2964	[−1.96, +1.96]	X
BDS(5)		8.9231	[−1.96, +1.96]	X

**Figure 4:**
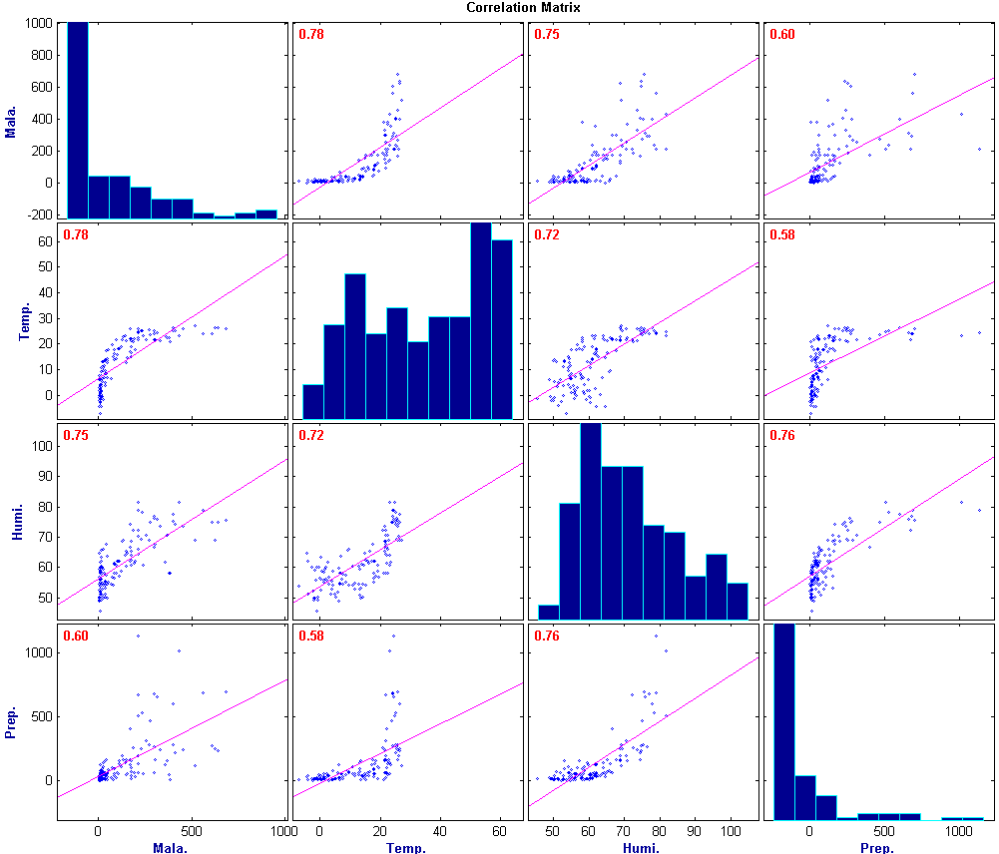
Correlation matrix of malaria and climate variable (Pearson’s correlation).

Results of multiple regression analysis show that the coefficient of determination $R^{2}$ of multiple regression model was 0.805, and the modified coefficient of determination $R^{2}$ was 0.753 and for the regression analysis model, F = 15.5. The significance probability was 4.57 × 10^−19^, and statistical significance did exist (see Figure 3a). However, according to Figure 1a, it is highly likely that there is coherency between data, and if this is the case, regression model drawn from regression analysis may be under the influence of multicollinearity. Thus, analysis was conducted based on malaria occurrences and each of the Pearson, Kendall and Spearman correlation coefficients; it revealed that each variable had a high correlation, from 0.52 between average temperature and humidity to 0.78 between average temperature and malaria (see Figure 4). Thus, there is clearly a strong correlation between each climate variable and there could be an error when building a regression model for malaria through multiple regression analysis.

### 4.2. PCA-Regression Analysis

**Figure 5:**
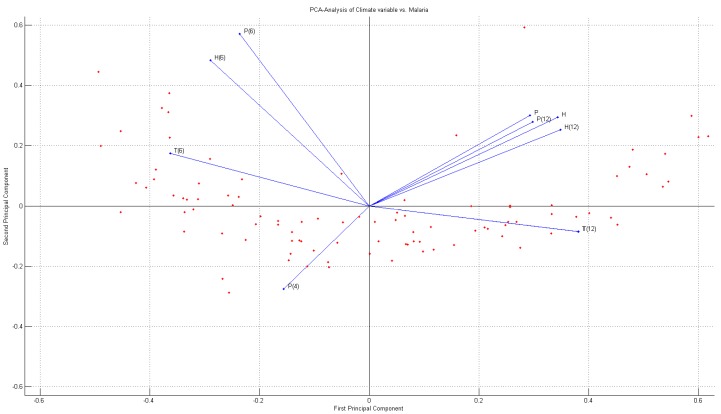
PCA-diagram.

To conduct principal component regression analysis between malaria occurrences and climate variables, principal component regression was conducted on average temperature, average humidity and precipitation. Principal components scores up to principal components 1, 2, and 3 were given—2.9698, 3.0490 and −0.6665, respectively. Principal components can be narrowed down to three with the condition of 80% cumulative contribution rate; the table of principal components analysis is shown in Figure 5.

Principal components were categorized into the following: first principal components were average temperature ($x_{1}$) and average humidity ($x_{5}$ , $x_{6}$); second principal components were average humidity ($x_{6}$ and precipitation $x_{7}$); and third principal components were average temperature ($x_{1}$) , average humidity ($x_{5}$ , $x_{6}$) and precipitation $x_{7}$). Principal component regression analysis can be conducted based on principal component analysis and principal component regression (PCA-regression) model of malaria occurrences is shown in Eqation (13):

(13) $$y=93.27-52.51z_{1}+4.77z_{2}-19.62z_{3}+6.58z_{1}^{2}-0.54z_{2}^{2}+5.28z_{3}^{2}\;z_{1}\text{: 1st principal component,}\;\begin{array}{l} z_{1}=0.38x_{1}-0.08x_{2}+0.05x_{3}-0.02x_{4}+0.46x_{5}\\ +0.37x_{6}+0.07x_{7}+0.09x_{8}-0.04x_{9}-0.69x_{10} \end{array}\;z_{2}\text{: 2nd principal component}\;\begin{array}{l} z_{2}=-0.36x_{1}+0.17x_{2}+0.08x_{3}-0.01x_{4}-0.14x_{5}\\ +0.32x_{6}+0.78x_{7}+0.14x_{8}-0.27x_{9}-0.02x_{10} \end{array}\;z_{3}\text{: 3rd principal component}\;\begin{array}{l} z_{3}=0.38x_{1}-0.08x_{2}+0.06x_{3}-0.001x_{4}-0.46x_{5}\\ +0.30x_{6}+0.15x_{7}-0.05x_{8}-0.10x_{9}-0.7x_{10} \end{array}$$

Results of malaria occurrence simulation based on the principal component regression model are compared in Figure 3. The coefficient of determination $R^{2}$ of principal components regression model was 0.743 and the modified coefficient of determination $R^{2}$ was 0.725. In the regression analysis model, F = 4.28 and significance probability was 3.4 × 10^−24^, thus statistical significance was present (See Figure 3b). Also, since each principal component is independent, the regression model can be established without the multicollinearity problem. Thus, principal components regression analysis is quite plausible for simulation of malaria occurrences. 

### 4.3. Validation of Malaria Model

**Figure 6:**
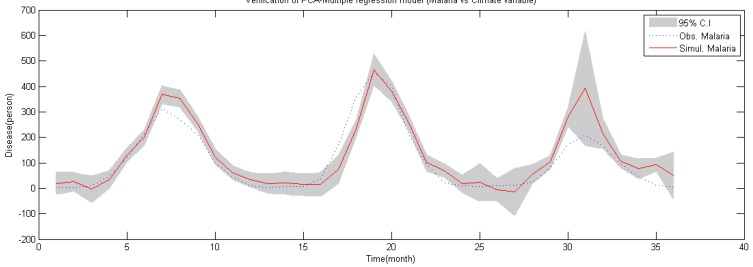
Validation of PCA-regression model (2009–2011).

To evaluate the suitability of malaria occurrence model calculated from PCA, evaluation of regression model was conducted using the malaria occurrence data from 2009–2011 and the results are shown in Figure 6. 

Results of evaluation using principal component regression analysis show that the coefficient of determination of PCA-regression model $R^{2}$ was 0.852 and the NRMSE (Normalized Root Mean Square Error) index, which shows the efficiency of the model suggested by Scott and Fred [54], was 0.117 and the RE (Relative Error; [36]) index was 0.026. It can be concluded that malaria occurrence time series simulated by the model does have significance with actual observation data. 

## 5. Malaria Occurrence under Climate Change

### 5.1. Climate Change Scenario

To simulate the condition of climate change using the malaria occurrence regression model as suggested in Section 4, future climate data is needed. In this study, future climate data was acquired through a climate change scenario and a climate model and then we analyzed the trend of malaria occurrences. For climate change scenario, future carbon dioxide concentration scenario was used as the boundary condition of the climate model and many different situations were assumed and applied. RCP (Representative concentration pathways) scenario, which will appear in the IPCC 5th assessment report released in 2013/2014, is the most studied scenario [55,56,57,58,59,60,61,62], and in this research, the RCP scenario was applied.

The RCP scenario is categorized into four different scenarios depending on the carbon dioxide reduction degree [57] (see Table 2). In this study, the SRES A1B scenario from the existing SRES AR4 scenario study [63], which counterparts RCP 4.5, and seems to conform with reality the most, was analyzed. Also, another RCP scenario study [64] presented that RCP 4.5 will most effectively reflect future climate in accordance with the present condition, so RCP 4.5 was applied. 

**Table 2:** RCP(Representative concentration pathways) scenario description.

Senarios	Description	CO density (ppm)
RCP 2.6	Peak in radiative forcing at ~3 W/m before 2100 year and then decline	490
RCP 4.5	Stabilization without overshoot pathway to ~4.5 W/m at stabilization after 2100 year	650
RCP 6.0	Stabilization without overshoot pathway to ~6 W/m at stabilization after 2100 year	850
RCP 8.5	Rising radiative forcing pathway leading to 8.5 W/m by 2100 year	1370

**Figure 7:**
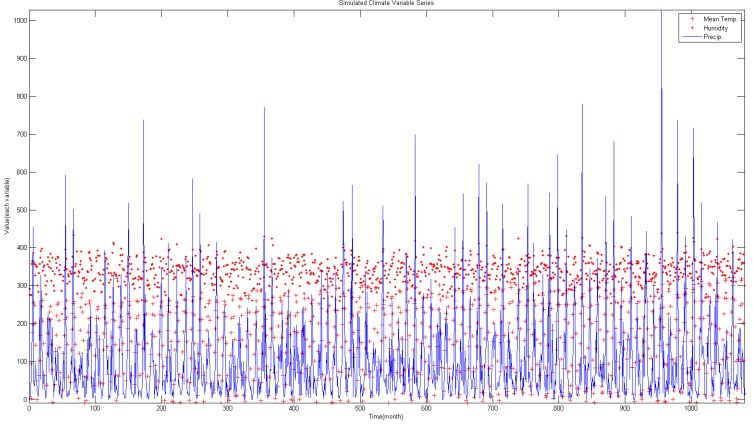
Simulated climate variable (2011–2100).

A numerical model which simulates the actual climate phenomenon using climate change scenario as the boundary condition was used for the climate model. The Korean government uses a local climate model HadGEM3-RA based on HadGEM2-AO model of Hadley Centre of British Met Office [65]. Monthly climate data from 2011–2100 according to RCP 4.5 climate change scenario and HasGEM3-RA climate model are shown in Figure 7. 

### 5.2. Future Malaria Simulation and Analysis

**Figure 8:**
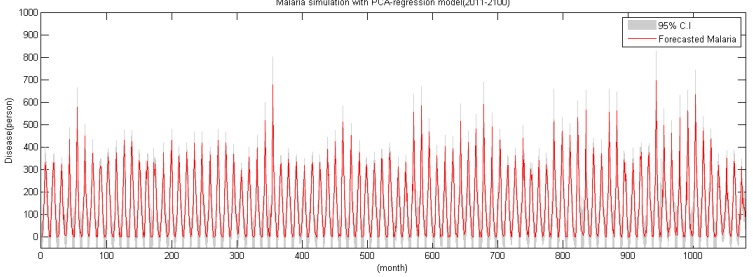
Simulated malaria time series (2011–2100).

The study simulated the future malaria occurrence using monthly climate data from 2011–2100 according to RCP 4.5 climate change scenario and the HadGEM3-RA climate model and malaria regression model. In the simulation data, 676 occurrences in July 2040 and 695 occurrences in July 2089 appeared to be the biggest incidents (Figure 8).

**Figure 9:**
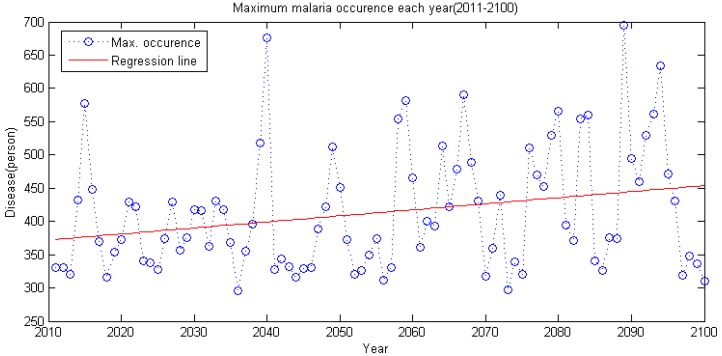
Annual maximum series of malaria (2011–2100).

There seems to be a general trend in accordance with the present state, but it shows a slight increase. When the annual maximum series is generated with the maximum malaria occurrence as a criterion, the trend becomes highly visible. As shown in Figure 9, simulation shows that annual maximum occurrence per year increases as time passes.

**Figure 10:**
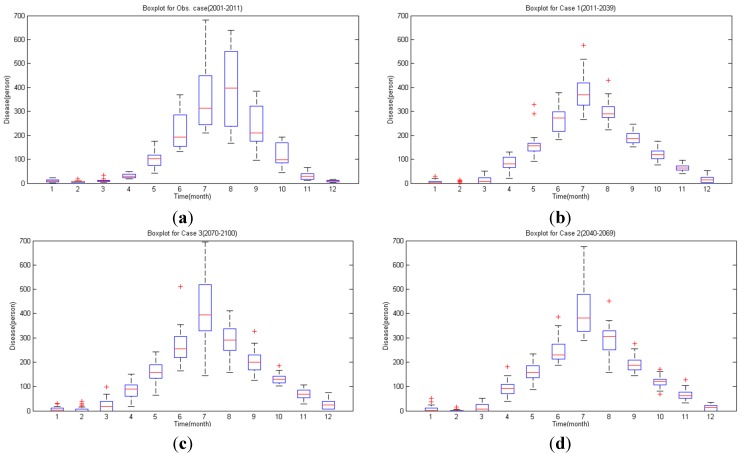
Malaria occurrence boxplot for each period 2001–2011 year (**b**) 2011–2039 year (**c**) 2040–2069 year (**d**) 2070–2100 year.

Also, there is some change in the annual occurrence trend. From 20010–2011, August was the month of maximum occurrence, but simulation data of 2011–2100 predicts July will be the month of maximum occurrence. In addition, compared to the spike of occurrence between June and August in the existing data, the occurrence will continually increase between April and July in the future (see the Figure 10).

**Figure 11:**
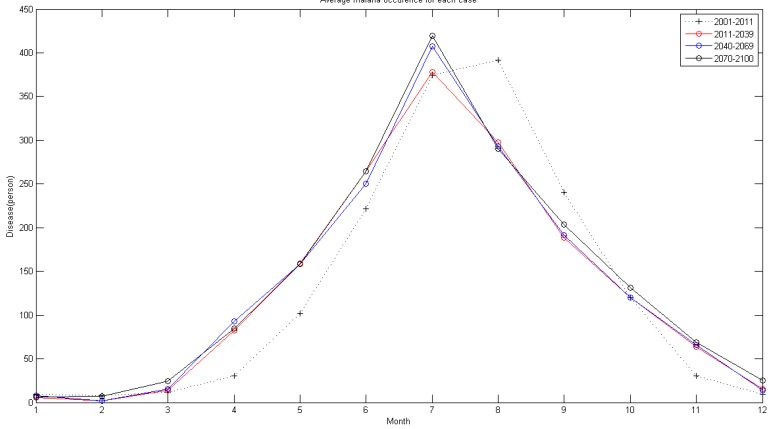
Monthly malaria occurrence plot for each period.

In Figure 11, the monthly average malaria occurrence is shown conclusively. Compared to the concentrated occurrence between May and August, simulation shows that the occurrence will start in April and will reach its maximum in July in the future. Thus, in the future, preparation for malaria outbreak must be executed earlier than the present guideline in which preparation starts after the rainy season in summer.

## 6. Conclusions

The study clarifies correlation between monthly climate data and malaria infection occurrence and establishes a regression model. Also, the future malaria occurrence up to year 2100 is simulated using future climate data generated for a climate change scenario and climate model and analysis on malaria occurrence time series. Results drawn from this study can be concluded as below:

1. Correlation between malaria occurrence and monthly average temperature, relative humidity and precipitation data is analyzed with time lag effect between malaria occurrence and climate variables using spectral analysis between each variable. A strong coherency between each climate variable data is clear, thus regression model is analyzed under the influence of multicollinearity. To resolve this issue, principal component regression analysis based on PCA is used to establish a regression model. Using the regression model, malaria infection occurrences from 2009–2011 are tested and coefficient of determination $R^{2}$ is 0.852, NRSE is 0.117 and RE is 0.026, which clearly accounts for malaria infection.
2. By applying climate data between 2011 and 2100 using the RCP 4.5 climate change scenario and the CNCM3 climate model to the regression model, future malaria occurrence is simulated. Analysis of simulated data shows the malaria occurrence trend in general will gradually increase. Also, in the future, the occurrence period will diminish and it shows an increase of malaria occurrence before the rainy season in summer; thus, adaptation in the malaria occurrence response plan of Korea is needed.
